# WinHAP2: an extremely fast haplotype phasing program for long genotype sequences

**DOI:** 10.1186/1471-2105-15-164

**Published:** 2014-05-30

**Authors:** Weihua Pan, Yanan Zhao, Yun Xu, Fengfeng Zhou

**Affiliations:** 1 School of Computer Science and Technology, University of Science and Technology of China, Hefei, Anhui 230027, P.R. China; 2 Anhui Province-MOST Co-Key Laboratory of High Performance Computing and Its Application, University of Science and Technology of China, Hefei, Anhui 230027, P.R. China; 3 Shenzhen Institutes of Advanced Technology, and Key Lab for Health Informatics, Chinese Academy of Sciences, Shenzhen, Guangdong 518055, P.R. China

**Keywords:** Haplotype phasing, Genotype, SNP, Long sequence, Parallel computing

## Abstract

**Background:**

The haplotype phasing problem tries to screen for phenotype associated genomic variations from millions of candidate data. Most of the current computer programs handle this problem with high requirements of computing power and memory. By replacing the computation-intensive step of constructing the maximum spanning tree with a heuristics of estimated initial haplotype, we released the WinHAP algorithm version 1.0, which outperforms the other algorithms in terms of both running speed and overall accuracy.

**Results:**

This work further speeds up the WinHAP algorithm to version 2.0 (WinHAP2) by utilizing the divide-and-conquer strategy and the OpenMP parallel computing mode. WinHAP2 can phase 500 genotypes with 1,000,000 SNPs using just 12.8 MB in memory and 2.5 hours on a personal computer, whereas the other programs require unacceptable memory or running times. The parallel running mode further improves WinHAP2's running speed with several orders of magnitudes, compared with the other programs, including Beagle, SHAPEIT2 and 2SNP.

**Conclusions:**

WinHAP2 is an extremely fast haplotype phasing program which can handle a large-scale genotyping study with any number of SNPs in the current literature and at least in the near future.

## Background

Single nucleotide polymorphisms (SNPs) are a kind of genomic variations that play an important role in many genetic analysis. Most eukaryotic genomes are diploid and it’s both technically difficult and time consuming to experimentally screen the sequence of alleles in contiguous SNP sites along each copy of the diploid chromosomes, which is called a haplotype. The two nucleotides/alleles for one locus in a chromosome are usually obtained as an unordered pair, which is called a genotype. A haplotype phasing problem is to infer haplotypes from genotypes. Although there are some other methods based on modern sequencing, such as Haplotype-resolved sequencing technology and HaploSeq method, can obtain haplotypes directly rather than computationally infer them, haplotype phasing costs much less money than these methods [1,2].

The existing methods for haplotype phasing problem can be classified into two major categories: combinatorial optimization algorithms [3,4] and statistical methods [5]. Combinatorial optimization algorithms focuses on finding the solution based on reasonable biological assumptions. Two models were considered: the Perfect Phylogeny Tree Model and the Maximum Parsimony Model. Maximum Parsimony Model assumed that the number of distinct haplotypes in natural populations was really small or the minimum among all feasible solutions [4]. The principle was firstly proposed by Wang and Xu who also presented a branch and bound algorithm under this principle to speed up the problem resolving [6]. Many other algorithms try to solve the problem based on this model using either SAT-based formulations or integer linear programming techniques. However, Maximum Parsimony Model has been demonstrated to be NP-complete [7] and APX-hard even in very restricted cases [8], which means precise solution can only be got with exponential time consuming. Therefore many approximate approaches were also proposed based on this model. Perfect Phylogeny Tree Model changed the haplotype phasing problem to a graph realization problem [3,9]. This model assumed that any SNP mutation happened just once in the whole evolutionary history. As perfect phylogeny trees are usually not unique for a given genotype set, the minimum perfect phylogeny haplotyping (MPPH) rule was proposed [7]. MPPH is a combination of Perfect Phylogeny Tree Model and the Maximum Parsimony Model. It tries to reconstruct a perfect phylogeny tree that consists of minimum number of unique haplotypes. Bafna et.al. proved MPPH problem to be NP-hard [10]. Although combinatorial optimization algorithms can do well in small datasets, the strong assumption and high time complexity holds back its application in larger datasets [11].

Compared with combinatorial optimization algorithms, statistical methods focus on estimating the haplotype frequencies according to certain statistical theories. An earlier method was EM algorithm [5]. It iteratively computes each haplotype’s frequency and estimate the new solution. Then the quality of the solution will be higher and higher. This algorithm worked well on a small data set, but its time cost increased sharply as it should enumerate all feasible solutions. In order to further reduce the computation time requirement, PLEM [12] and GERBIL [13] use partition-ligation strategy for speed-up. And BPPLEM replaces the uniform strategy with non-uniform strategy based on linkage disequilibrium (LD) [14]. In addition, some other statistical theories have also been used to infer the haplotypes, such as Bayesian and MCMC [15]. Although statistical methods can process larger datasets than combinatorial optimization algorithms, they usually need to consider lots of feasible haplotypes, which require large amount of storage.

With the recent innovations in high-throughput gene chip technologies, huge amount of genotype data was produced, leading to a new challenge of handling the large-scale datasets for the haplotype phasing problem. many methods based on Hidden Markov Model (HMM) have been proposed recently, such as Beagle [16], HAPI-UR [17], SHAPEIT1 [18] and SHAPEIT2 [19]. This kind of methods firstly yields feasible haplotypes randomly. Then it iteratively builds HMM according to current haplotypes and gets new haplotypes based on this HMM. Our experiments have shown that while they can get accurate haplotype results for datasets with a large number of homologous sequences, they can’t do well for datasets with small number of long genotype sequences which are very common. That’s because statistical methods need more information to refer to compared with other methods. In addition, some methods combine statistical theories and combinatorial optimization rules. Although it’s impossible to get exact solution based on combinatorial optimization rules for large scale datasets, they are used in some steps of algorithms to help to improve the precision of approximate solutions. For example, 2SNP algorithm finds the most relevant allele for a specific allele by building a phylogeny tree [20].

We proposed the WinHAP algorithm [21] by combining probability statistic and combinatorial optimization [21]. WinHAP significantly improved the speed of haplotype phasing, while achieving similar or better overall accuracy compared with the other existing programs. But days are still needed for WinHAP to screening the millions of SNPs in the human genome. We further improve the program's running speed and memory efficiency by using the following two strategies. Firstly, a divide-and-conquer strategy is utilized to solve the challenge of huge computer memory required by the existing algorithms. The basic idea is to screen the long chromosomes for haplotypes within the consecutive 1,000-SNP segments. Thus, the memory need of the algorithm is only related with one segment and no longer increases as length of sequences. Secondly, the OpenMP parallel computing mode is implemented to utilize all the computing power in a multi-core computer cluster. The haplotype phasing performance of WinHAP version 2 (WinHAP2) is discussed in the following sections.

WinHAP2.0 software package is available at http://staff.ustc.edu.cn/~xuyun/winhap/index.htm. We have also uploaded the source code, manual, materials and example datasets onto it. As of now, WinHAP software packages have been downloaded for at least dozens of times and used by biologists from different organizations and institutes around the world, such as Max Planck Institute for Molecular Genetics, Dahlem Centre for Genome Research and Medical Systems Biology, Alacris Theranostics GmbH in Germany, University of Medical Sciences in Poland, Jiangxi Agricultural University and University of Science and Technology of China. Particularly, some users from Huazhong Agricultural University want to process very long sequences with more than 1 million SNPs on personal computers which cannot be done by WinHAP1.0 and nearly all the existing tools except for WinHAP2.0.

## Implementation

The input to WinHAP2 consists of *n* genotype vectors, each with *m* coordinates corresponding to *m* SNPs. Each genotype vector can be phased to two haplotype vectors with value in {0, 1}, where ‘0’ represents the major allele and ‘1’ the minor allele. A haplotype vector is represented as a string of the alphabet {0, 1}. A genotype vector is represented as a string of alphabet {0, 1, 2, ?}, where ‘0’ and ‘1’ represent the homozygous SNP {0, 0} and {1, 1}, respectively, ‘2’ is a heterozygous SNP {0, 1}, and ‘?’ is a missing SNP.

### Divide-and-conquer strategy

**Figure 1:**
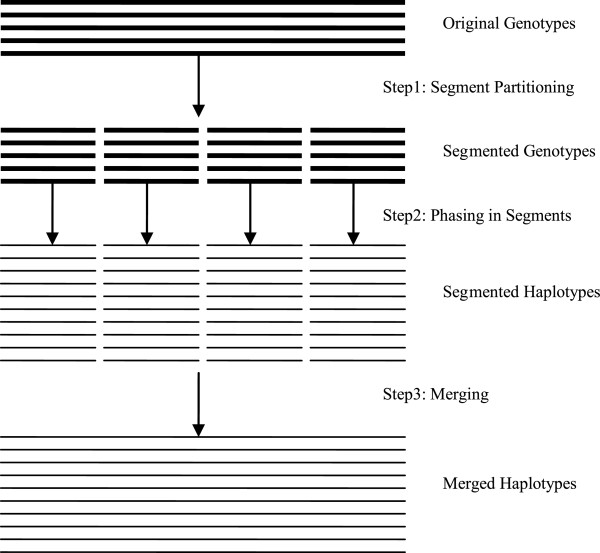
**Framework of WinHAP2.** Procedure of WinHAP2 with divide-and-conquer strategy. Explanation see text in this section.

WinHAP2 utilizes the divide-and-conquer strategy to phase long sequences. The WinHAP algorithm infers haplotypes from the neighboring SNPs, and the comparison with the other programs suggests that little association exists between a pair of remote SNPs. So a segment with fixed number of consecutive SNPs (1,000 by default) is sufficient for inferring haplotypes.With divide-and-conquer strategy, WinHAP2 consists of three phases. In first step, the genotypes are partitioned into segments. In second step, all segments are phased by WinHAP respectively. In last step, the results of all segments are merged into a whole result. Figure 1 shows the overall framework.

In the first step, we partition genotype datasets sequentially into segments, each of which has the same size*s*. It should be mentioned here that the value of *s* must be neither too large nor too small. Larger *s*is, more memory is cost and then partition will be less meaningful. On the other hand, if *s*is too small, precision of algorithm will be affected. Because it’s possible that there’s no ‘2’ in one segment, which will make it difficult to merge the haplotype result of that segment with others’. Based on our experiments, *s* should be larger than 1,000 sites, while the upper limit of *s*is related with the memory of computer used. For the last segment, as we know, the size of it is less than or equal to *s*. If it is smaller than 1,000 sites, we merge it to the last but one segment.

In the second step, all segments are phased by WinHAP respectively. WinHAP has three phases: In the first phase which is called simplified 2SNP algorithm, the initial haplotype results are obtained. In the second phase, scalable sliding windows are used to correct some errors in first phase. In the third phase, maximum parsimony principle is used to improve the quality of results further. Two points should be mentioned here. Firstly, in the second phase, precision of the sites near edge is lower than others’ because they can only be covered by sliding windows from one side. Number of this kind of sites becomes much larger in WinHAP2 because each segment has edges. This problem is solved in the third step of WinHAP2. Secondly, the third phase can get better results in WinHAP2, because maximum parsimony principle is not suitable for very long sequences and segmenting makes the sequences shorter.

In the final step, the results of all segments are merged into a whole result. To ensure the precision of the SNPs at the edge of each segment, a merging strategy is proposed. It’s described in detail in section Method.

### Parallelization

Due to its nature of local calculation, and its large data size, an OpenMP parallel computing mode is adopted for the time-consuming step of haplotype phasing including step 2 and step 3. As phasing in each segment has no relationship with other segments, parallelization of step 2 is comparatively easy. We just distribute one or several segment to one thread and each thread gets the result respectively. However, parallelization of step3 is harder. For merging is a process involving all segments, how to distribute the assignment to each thread is the key point. To eliminate the relationship between segments, we divide the process into 3 phases. Firstly, we cut the right edge of each segment except last one and save it in file, which can be done by each thread respectively. Secondly, each thread merge the right edge of the former segment and later segment and save the merged each segment. Finally, one thread put all merged segments together, which is hardly time-consuming.

### Time complexity of WinHAP2

Now, let’s analyze the time complexity of WinHAP2. In the first step, WinHAP2partitions genotypes into segments, which takes *0*(*nm*) time since we have *n* genotypes each with *m* SNPs. In the second step, each segment is phased respectively by WinHAP. According to the analysis in the previous paper [21], WinHAP takes *0*(*n*^2^*m*). So if we use *p* computing cores, it takes *0*(*n*^2^*m*/*p*). In the last phase, our algorithm merges the results of all segments into a whole result using scalable sliding window. For each two adjacent segments, *l*_max_-2 windows are needed. And the computation in one window takes *0*(*n*) time. So the last phase takes *0*(*nk*/*p*), where *k* denotes the number of segments and *p* denotes the number of computing cores. Thus, our algorithm takes *0*(*nm* + *n*^2^*m*/*p* + *nk*/*p*)~ *0*(*n*^2^*m*/*p*) in total time.

## Results and discussion

### Datasets

As the performance of WinHAP processing comparatively small datasets (<1000 SNPs) like ACE [22], 5q31 [23] and CFTR [24] has been showed in the previous paper [21], only large scale datasets with more than 10,000 SNPs are tested in this paper. However, public real datasets of this scale are very few. So we employ one real dataset, one simulated dataset which is generated by randomly pairing real haplotypes, and simulated datasets of different scale from ‘ms’ software.

### HapMap real dataset

Firstly, we compared the performances of WinHAP2 and the other Haplotype Phasing programs on the real dataset from International HapMap Project which aims to develop a haplotype map ofthe human genome [25]. This dataset consists of 44 pedigrees (father, mother and child), each genotyped at 36,258 SNPs in the 20th chromosome of human. We choose the genotypes of 44 fathers and 44 mothers to get 88 unrelated genotype sequences. As SHAPEIT2 is a newest algorithm for large scale datasets, we want to compare WinHAP2with it. The input of SHAPEIT2 includes recombination rate of each SNP, and HapMap shows a table which includes recombination rate of most SNP in human chromosome. However, there are still some SNPs of this real datasets are not in this table. So we have to choose 32,458 SNPs from original dataset. So it’s a dataset comprising of 88 unrelated genotype sequences, each of which has 32,458 sites.

### HapMap simulated dataset

We further tested the algorithms with another dataset from HapMap International HapMap Project. It’s a dataset of 120 real haplotype sequences, each of which has 63,810 SNPs in the 20th chromosome of CEU (Utah residents with ancestry from northern and western Europe). We generated the genotype datasets by randomly pairing two haplotypes. To let the simulated genotype dataset be similar to real ones, we only generate 40 sequences. So it’s a dataset comprising of 40 unrelated genotype sequences, each of which has 63,810 sites.

### “*ms*” dataset

We use well-known Hudson’s software “ms” [26] to generate simulated genotype sets with *N* = 50, *N* = 100, *N* = 200, *N* = 500 and *M* = 10,000, *M* = 20,000, *M* = 50,000, *M* = 100,000, *M* = 1,000,000. Here *N* means the number of sequences and *M* means the length of sequences. The parameter“*θ*” is set to 5.0. The recombination parameters “*ρ*” and “nsites” are set to 100 and 2501 respectively.

### Measurement criteria of phasing accuracy

Usually, the *individual error rate* (IER) [15] and the *switch error rate* (SER) [13] are used to evaluate the performance of phasing algorithms [27,28]. IER is defined as the percentage of individuals whose genotypes are incorrectly resolved and SER is defined as the ratio between the numbers of switch errors and all the heterozygous loci. The value of IER usually increases along with the increase of genotype length. When the number of SNPs is large enough, the IER value of almost all haplotype phasing approaches is close to 100%. In our experiments, the number of SNPs in all datasets is larger than 10,000, and IER is meaningless for datasets of this scale. So we just use switch error rate (SER) to evaluate the performance of WinHAP2 in this paper.

We compared our algorithm with three existing programs including SHAPEIT2 [19], Beagle [16] and 2SNP [20]. Other algorithms are not tested for either of the following three reasons: (1) can’t produce the results within reasonable time; (2) cannot handle the missing SNPs; (3) can’t process such long sequence.

### Validation on HapMap real dataset

We run the 2SNP, Beagle, SHAPEIT2 and WinHAP2 on a HapMap real dataset averaged over 100 independent runs. The dataset comprises of 88 unrelated genotype sequences, each of which has 32,458 SNPs. 2SNP and SHAPEIT2 were both run with the default settings. The parameter “nsample” of Beagle was set to 4 and we randomly generated the parameter “seed” in every independent running.

The performance of various phasing programs on this HapMap real dataset was shown in Table 1. From the result, we can see that although the error rate of WinHAP2 is not the best, it’s similar to others’. But the running time and memory consumption of WinHAP2 are both much lower than others’. While SHAPEIT2gets the lowest error rate, its running time is about 60 times and its memory consumption is 30 times than WinHAP2. In addition to that, the use of SHAPEIT2 has some limit which means that it needs the recombination rate of each SNP, but not all SNPs’ recombination rate can be got. Beagle’s running time is 3 times and memory consumption is more than 100 times than our method. The precision and memory consumption of 2SNP are both the worst among the programs, and its running time is nearly 30 times than our algorithm’s.

**Table 1:** Results on HapMap real dataset

**Software**	**SER**	**Time (s)**	**Memory**
**2SNP**	0.049	892.70	1.0GB
**Beagle**	0.032	121.30	396.5 MB
**SHAPEIT2**	**0.024**	1816.20	91.4 MB
**WinHAP2**	0.039	**32.00**	**3.2 MB**

Bold data means the best result in terms of the corresponding evaluation standard.

### Validation on HapMap simulated dataset

To test the performance of WinHAP2 on datasets with longer sequences, we run 2SNP, Beagle and WinHAP2 on HapMap simulated dataset. As SHAPEIT2 need the information like recombination rate of each SNP and sex of each individual, it cannot be run on simulated datasets. We constructed a dataset of 40 haplotypes with no missing data from 120 experimentally identified disease haplotypes. Each haplotype has 63,810 SNPs. To ensure the objection of the test, we repeat the sampling and test for 100 times. The parameter “nsample” of BEAGLE was set to 4. All the other parameters were set to the default values.

Table 2 gives the accuracies, running times and memory consumption of the algorithms. Through WinHAP2has higher SER than Beagle, the running speed is about 3 times than Beagle’s and memory consumption is only about one thirtieth of Beagle’s. The precision, running time and memory consumption of 2SNP are all the worst among the algorithms.

**Table 2:** Results on HapMap simulated dataset

**Software**	**SER**	**Time (s)**	**Memory**
**2SNP**	0.034	1342.10	2.2GB
**Beagle**	**0.022**	92.89	622.2 MB
**WinHAP2**	0.028	**37.92**	**2.4 MB**

Bold data means the best result in terms of the corresponding evaluation standard.

### Validation on ‘ms’ simulated datasets

To test the performance of WinHAP2 on different size of datasets, we run 2SNP, Beagle and WinHAP2 on ‘ms’ simulated datasets.

The performance of various phasing algorithms on this ‘ms’ simulated dataset was shown in Table 3. From the result, we can see that SERs of WinHAP2 are similar with and sometimes the same with Beagle’s. The running speed of our algorithm is 3 to 10 times than Beagle’s. In addition, Beagle cannot process 200 sequences with more than 100,000 SNPs or 500 sequences with more than 50,000 SNPs on our machine because of memory overflow. 2SNP cannot process 100 sequences with more than 100,000 SNPs for the similar reason. WinHAP2 can process sequences with 1,000,000 SNPs using only 12.8 MB memory.

**Table 3:** **Results on** ‘**ms**’ **simulated datasets**

**Data sets**	**Methods**	**SER**	**Time (s)**	**Memory**
#genotype = 200	2SNP	0.037	87.8	197.0 MB
#SNPs = 10,000	Beagle	**0.020**	121.4	481.7 MB
	WinHAP2	**0.020**	**14.5**	**5.1 MB**
#genotype = 200	2SNP	0.035	2472.0	1.7GB
#SNPs = 50,000	Beagle	**0.019**	582.8	617.6 MB
	WinHAP2	0.024	**69.9**	**5.1 MB**
#genotype = 500	2SNP	0.023	8404.0	1.7GB
#SNPs = 50,000	Beagle	Out of memory		
	WinHAP2	**0.012**	**340.5**	**12.8 MB**
#genotype = 500	2SNP	Out of memory		
#SNPs	Beagle	Out of memory		
=1,000,000	WinHAP2	**0.012**	**9013.6**	**12.8 MB**

Bold data means the best result in terms of the corresponding evaluation standard.

**Figure 2:**
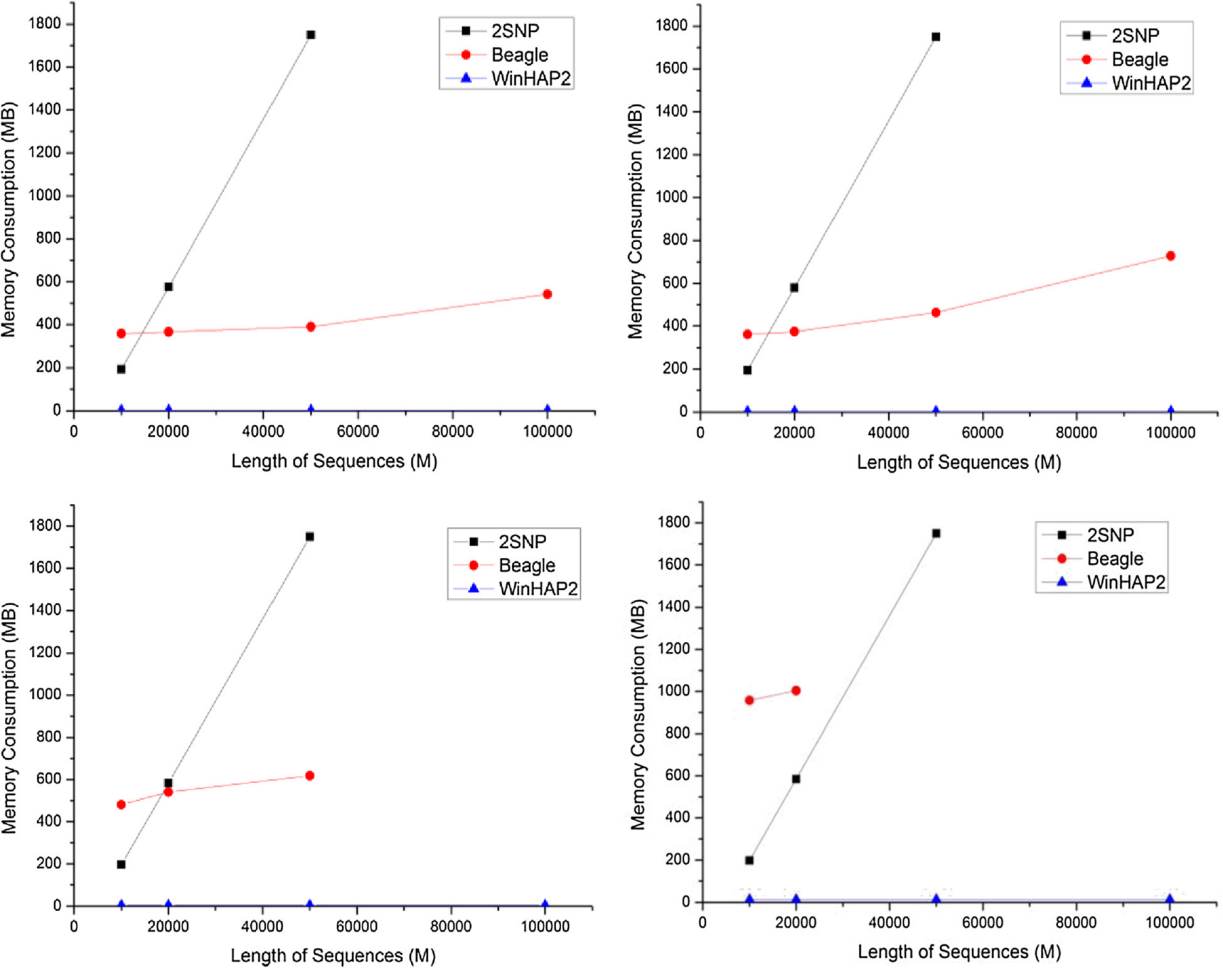
**Comparison of memory consumption variation trend of WinHAP2 with other methods.** TopLeft: The number of the sequences in the datasets is 50; TopRight: The number of the sequences in the datasets is 100; DownLeft: The number of the sequences in the datasets is 200; DownRight: The number of the sequences in the datasets is 500. As Beagle and 2SNP cannot get the result for some large datasets in our personal computer, the curves about the two programs lack of some points.

Figure 2 shows that the memory requirement of WinHAP2 received minor changes for test datasets with different numbers of SNPs. By analyzing the WinHAP2 algorithm, we may hypothesize that its memory requirement per genotype should be a constant, and actually the values (Memory)/(#Genotype) are 0.0364, 0.0600, 0.0255, 0.0255, 0.0256, and 0.0256 for the six datasets in Tables 1, 2 and 3, respectively. Most of current large-scale genotyping studies sampled fewer than tens of thousands of individuals [29], and their memory requirement using WinHAP2 is estimated to be ~330 MB. So WinHAP2 can handle a large-scale genotyping study with any number of SNPs in the current literature and at least in the near future.

### Performance of WinHAP2 using different sizes of segments

**Figure 3:**
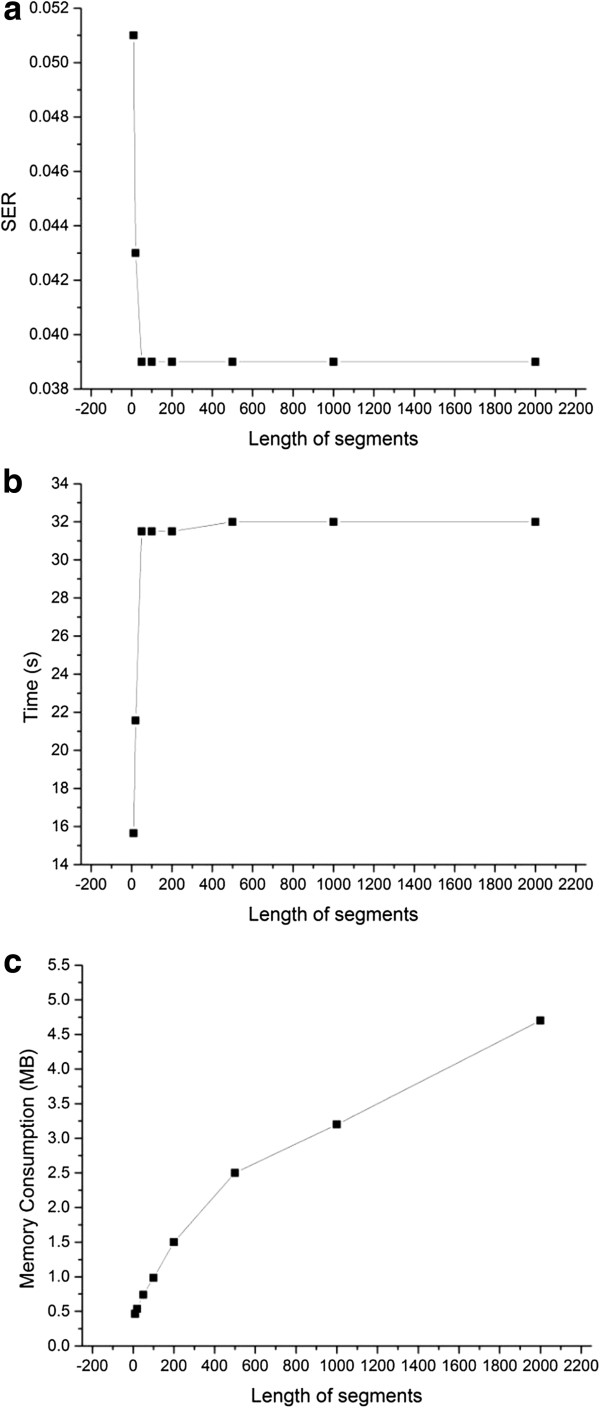
**Performance of WinHAP2 using different sizes of segments. (a)** The relationship between switch error rate and the length of segments; **(b)** The relationship between running time and the length of segments; **(c)** The relationship between memory consumption and the length of segments. Explanation see text in this section.

To test how the different sizes of segments affect the accuracy and computation load, we run WinHAP on the HapMap real dataset using different sizes of segments.Figure 3 shows that the accuracy will reduce extremely when the segments are too short. At the same time, the longer the segments are, the more running memory is used. Therefore, the moderate size of segments should be chosen.

### Performance of parallelization

**Figure 4:**
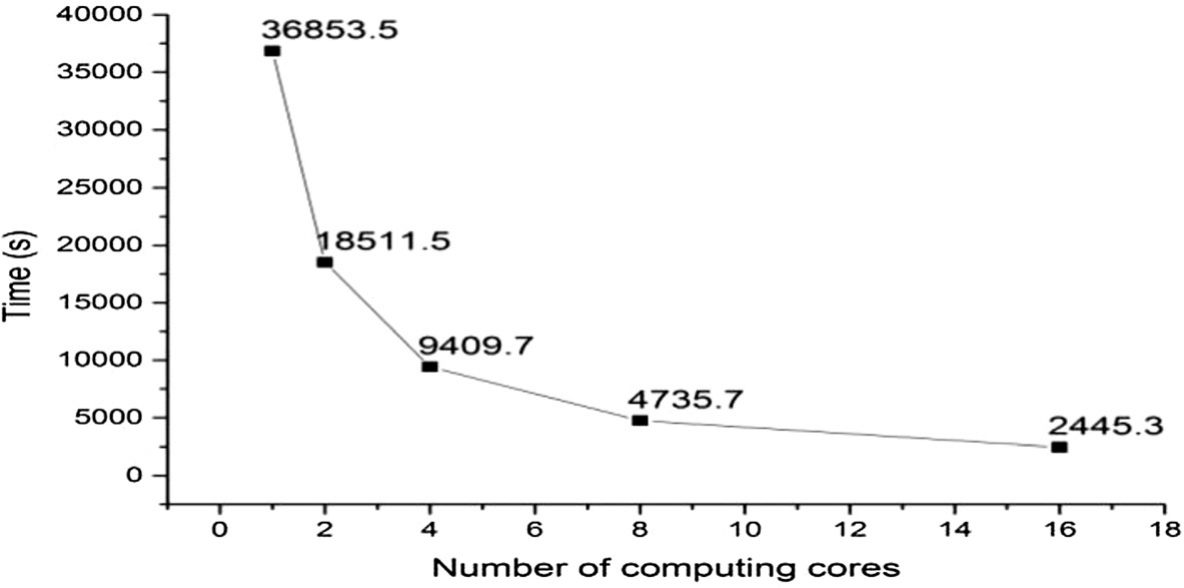
**Performance improvements of parallelized WinHAP2.** Runtimes of parallelized WinHAP2 using 1, 2, 4, 8, and 16 computing cores. The program was run on a Linux server with 16 800 MHz computing cores and 23.6GB memory. The dataset is a ms dataset which has 500 genotypes with 1,000,000SNPs.

We further tested the running speed of parallelized WinHAP2 on a server with 16 computing cores. The program was run on a Linux server with 16 800 MHz computing cores and 23.6GB memory. The dataset is a ms dataset which has 500 genotypes with 1,000,000 SNPs. Figure 4 shows that WinHAP2 using 16 computing cores was more than 15 times faster than the single threaded WinHAP2. Considering the running speed of WinHAP2 with single processor has already been several or dozens of times than other high performance algorithms which has been above showed, the parallelized WinHAP2 with 16 computing cores is over 40 times faster than Beagle, and even 50 to 200 times faster than SHAPEIT2 and 2SNP.

## Conclusions

With the development of large-scale sequencing technologies, a large amount of genotype data is being generated. Algorithms for large-scale haplotype phasing are needed. Most of existing programs cannot process extremely large datasets because of either space limit or time consumption.

In this article, we introduced a computer program, WinHAP2, which achieves significant improvements in running speed and memory requirement, with better or comparable precision, for the haplotype phasing problem. WinHAP2 can handle a large-scale genotyping study with over 1,000,000 SNP sites, which is beyond the capability of the other existing programs.

## Method

### Merging strategy

After we get the haplotype results of all segments, we must merge them to obtain the whole haplotype result. Here, we introduce how to merge the haplotype result of the *f*^th^ segment with that of the (*f* + 1)^th^ segment. Let $\langle h_{i}^{f}\overset{—}{h_{i}^{f}}\rangle$ and $\langle h_{i}^{f+1}\overset{—}{h_{i}^{f+1}}\rangle$ denote the haplotype results of *i*^th^ genotype in *f*^th^ segment and (*f* + 1)^th^ segment, respectively. Then, for *i*^th^ genotype vector, merging is a process to determine the pairing of $\langle h_{i}^{f}\overset{—}{h_{i}^{f}}\rangle$ and $\langle h_{i}^{f+1}\overset{—}{h_{i}^{f+1}}\rangle$. For example, if $\langle h_{i}^{f}\overset{—}{h_{i}^{f}}\rangle$ = 〈0000001001〉 and $\langle h_{i}^{f+1}\overset{—}{h_{i}^{f+1}}\rangle$ = 〈0100000001〉, we must determine whether the merged result $h\bar{h}$ is 〈00000010000100100001〉 or 〈00000000010100101000〉. The scalable sliding window described in the previous paper [21] is used to complete it.

Here, we assume that the length of the scalable sliding window ranges from *l*_min_ to*l*_max_, which can be different from the value in the previous paper [21]. At the beginning, the left edge of the window locates at the last site but *l*_max_-1 of *f*^th^ segment and the right edge locates at the first site of (*f* + 1)^th^ segment. Then the window slides from left to right and ends when the left edge locates at the last site of the *f*^th^ segment. At each position, if the window only covers the ‘2’s from one segment, this position should be given up and the window slides to next position. Otherwise, we get the most potable haplotype result of each genotype and compute the weight that corresponds to it. The process and definition of the weight was described in the previous paper [21]. During the sliding of the window, we always save the current best weight and the result corresponding to it. At last, the two segments are merged using the result with the best weight.

However, there’re some exceptional cases that the method upper-mentioned cannot be used. Here, we define another window called scope window *W*_*f*_ to explain them. *W*_*f*_ represents the whole scope that scalable sliding window slides in, thus its left edge locates at the (*l*_max_-1)^th^ site from right edge of *f*^th^ segment and the right edge at the (*l*_max_-1)^th^ site of (*f* + 1)^th^ segment. Let $g_{i}^{f}$ and $g_{i}^{f+1}$ denote the *i*^th^ genotype of *f*^th^ segment and the *i*^th^ genotype of (*f* + 1)^th^ segment, respectively. Then three exceptional cases are described as follows:

Case1: For either $g_{i}^{f}$ or $g_{i}^{f+1}$, there’s no ‘2’ in *W*_*f*_.

Case2: For both $g_{i}^{f}$ and $g_{i}^{f+1}$, there’s no ‘2’ in*W*_*f*_.

Case3: For either $g_{i}^{f}$ or $g_{i}^{f+1}$, there’s no ‘2’ .

**Figure 5:**
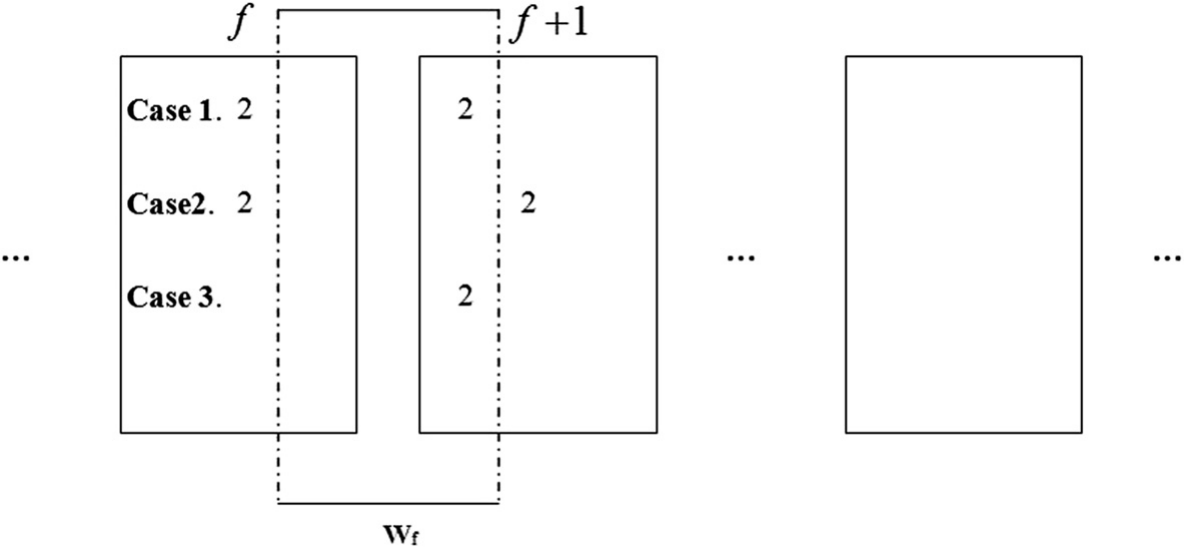
**Three cases in which scalable sliding window cannot merge the segments.** Explanation see text in this section.

For the 3 cases which are showed in Figure 5, scalable sliding window cannot cover the last ‘2’ in *f*^th^ segment and the first ‘2’ in (*f* + 1)^th^ segment at any position. Therefore, paring of $\langle h_{i}^{f}\overset{—}{h_{i}^{f}}\rangle$ and $\langle h_{i}^{f+1},\overset{—}{h_{i}^{f+1}}\rangle$ cannot be determined by sliding window. For case1 and case2, we use simplified 2SNP algorithm which is described in the previous paper [21] to determine the phasing result of the last ‘2’ in *f*^th^ segment and the first ‘2’ in (*f* + 1)^th^ segment. For case3, as it’s impossible to determine the paring, we just choose one randomly. Although, as we know, there is probability of 50 percent to get a wrong choice, it hardly affects the precision because the possibility of case3’s occur is extremely low.

## Availability and requirements

**Project name**: WinHAP Software

**Project home page**: http://staff.ustc.edu.cn/~xuyun/winhap/index.htm

**Operating systems**: Linux(32bit) or Linux(64bit)

**Programming language**: C, C++

**Other requirements**: none

**Any restrictions to use**: none

## Competing interests

The authors declare that they have no competing interests.

## Authors’ contributions

WP designed and implemented the software. WP and YX designed some strategies in the software. WP and FZ drafted the manuscript. YZ tested the software. YX and FZ provided feedback on the software development and manuscript. All authors read and approved the final manuscript.
